# Supplementary material for: Qualitative and Quantitative Analysis of Secondary Metabolites in Morphological Parts of Paulownia Clon In Vitro 112® and Their Anticoagulant Properties in Whole Human Blood
Source: Molecules. 2022 Feb 1;27(3):980. doi: 10.3390/molecules27030980 (PMC8840654; doi:10.3390/molecules27030980)
Supplement: Supplementary file 1 [file molecules-27-00980-s001.zip › molecules-1532305-supplementary.pdf]

molecules

MDPI

*Supplementary Materials*

# Qualitative and Quantitative Analysis of Secondary Metabolites in Morphological Parts of Paulownia Clon In Vitro 112® and Their Anticoagulant Properties in Whole Human Blood

**Anna Stochmal [1], Barbara Moniuszko-Szajwaj [1], Jerzy Zuchowski [1], Łukasz Pecio [1], Bogdan Kontek [2], Malgorzata Szumacher-Strabel [3], Beata Olas [2,*] and Adam Cieslak [3]**

1 Department of Biochemistry and Crop Quality, Institute of Soil Science and Plant Cultivation—State Research Institute, Czartoryskich 8, 24-100 Puławy, Poland; asf@iung.pulawy.pl (A.S.); bszajwaj@iung.pulawy.pl (B.M.-S.); jzuchowski@iung.pulawy.pl (J.Z.); lpecio@iung.pulawy.pl (Ł.P.).
2 Department of General Biochemistry, Faculty of Biology and Environmental Protection, University of Lodz, 90-236 Łódź, Poland; bogdan.kontek@biol.uni.lodz.pl.
3 Department of Animal Nutrition, Poznań University of Life Sciences, Wołyńska 33, 60-637 Poznań, Poland; mstrabel@joy.up.poznan.pl (M.S.-S.); adamck@joy.up.poznan.pl (A.C.).
* Correspondence: beata.olas@biol.uni.lodz.pl

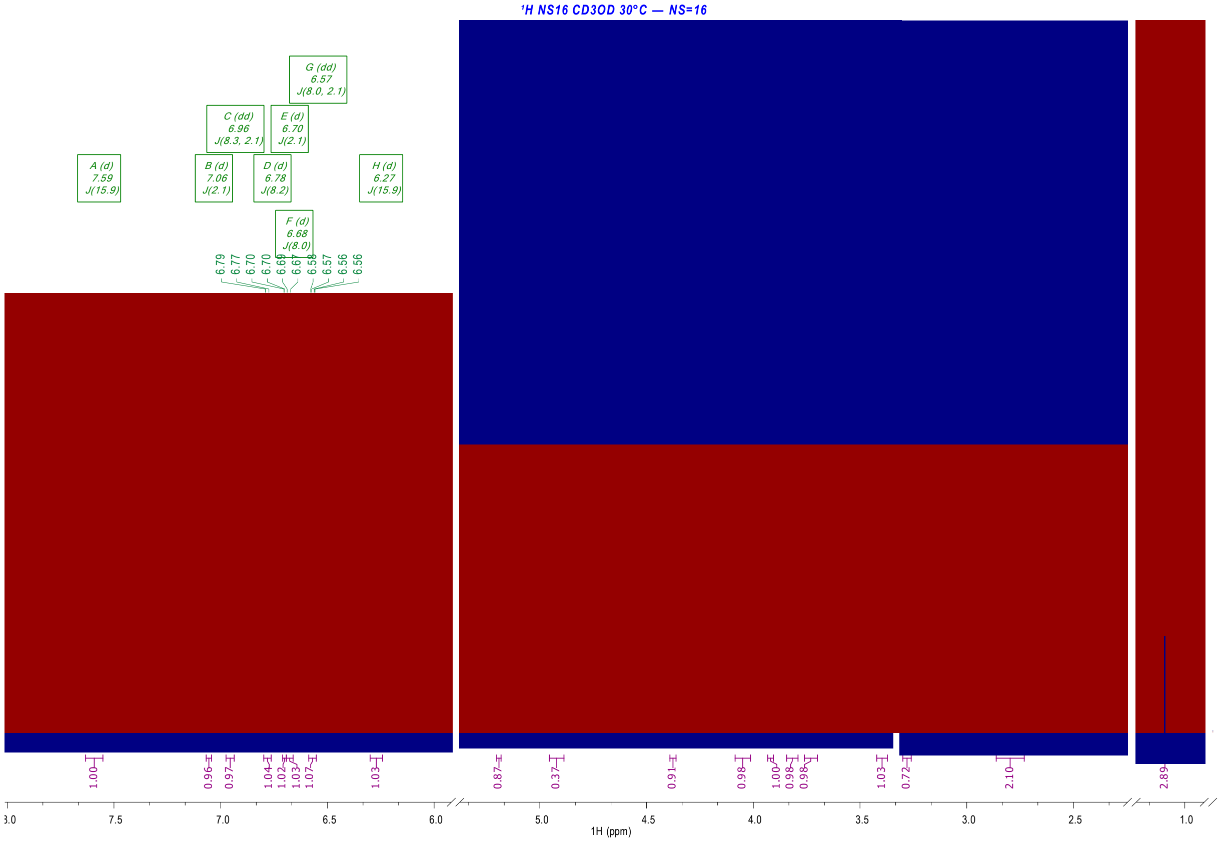


**Figure S1.** ¹H-NMR spectrum of verbascoside (500 MHz, MeOH-$d_4$, 30 °C).

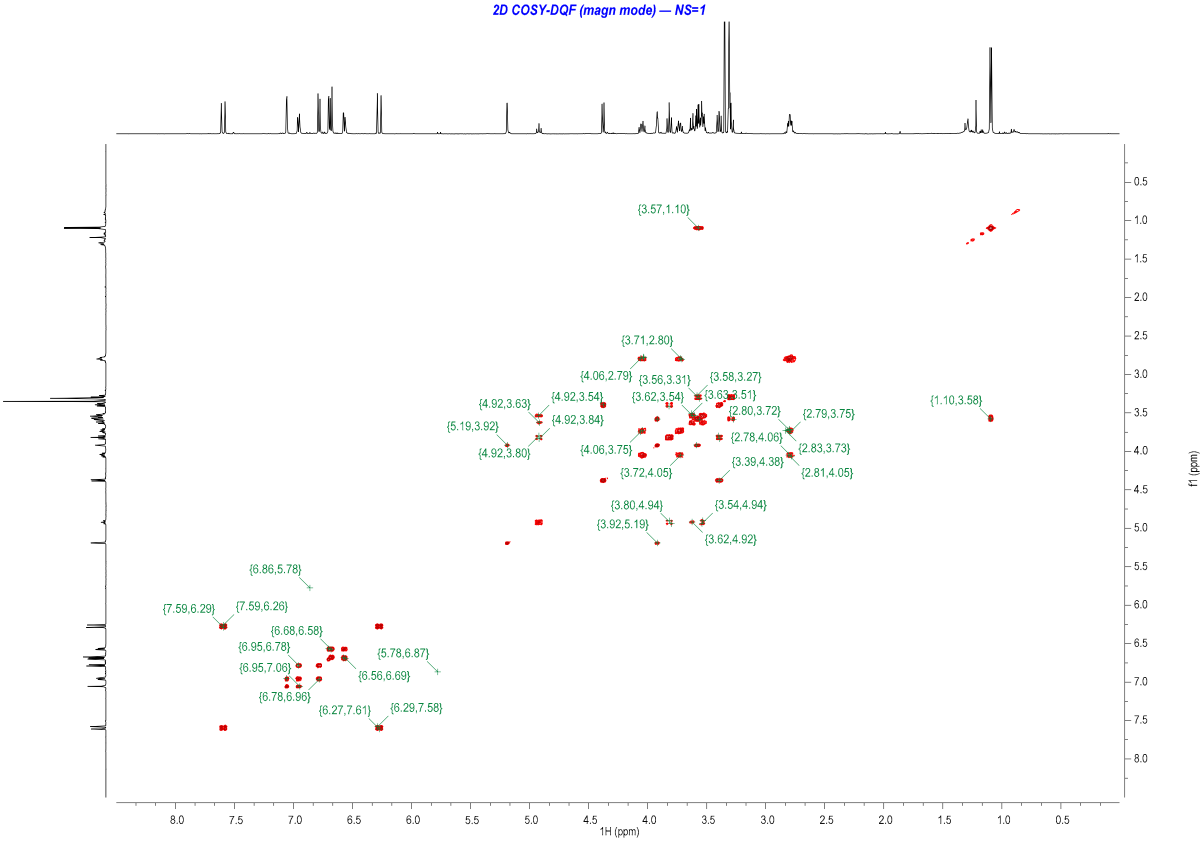


**Figure S2.** $^{1}H$-$^{1}H$ DQF-COSY NMR spectrum of verbascoside (500 MHz, MeOH-$d_4$, 30 °C).

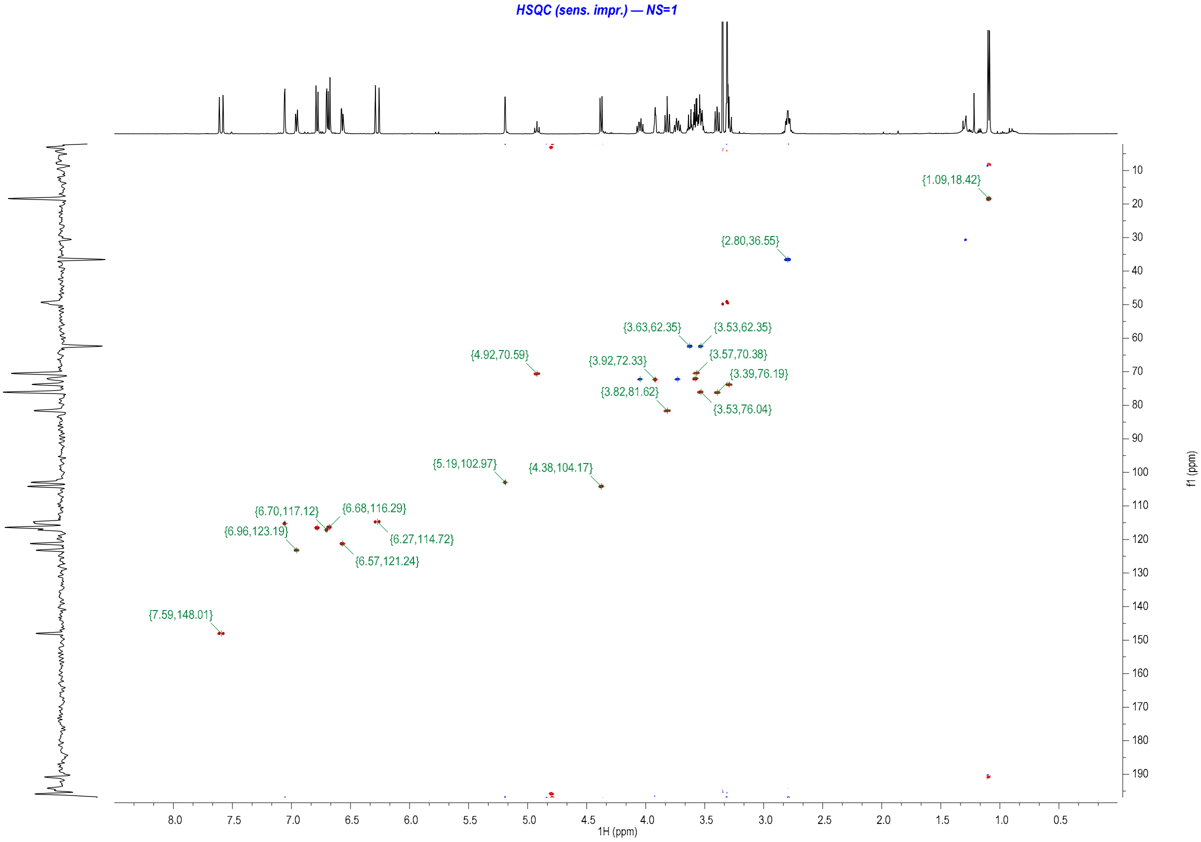


**Figure S3.** $^{1}H$-$^{13}C$ HSQC NMR spectrum of verbascoside (500/125 MHz, MeOH-$d_4$, 30 °C).

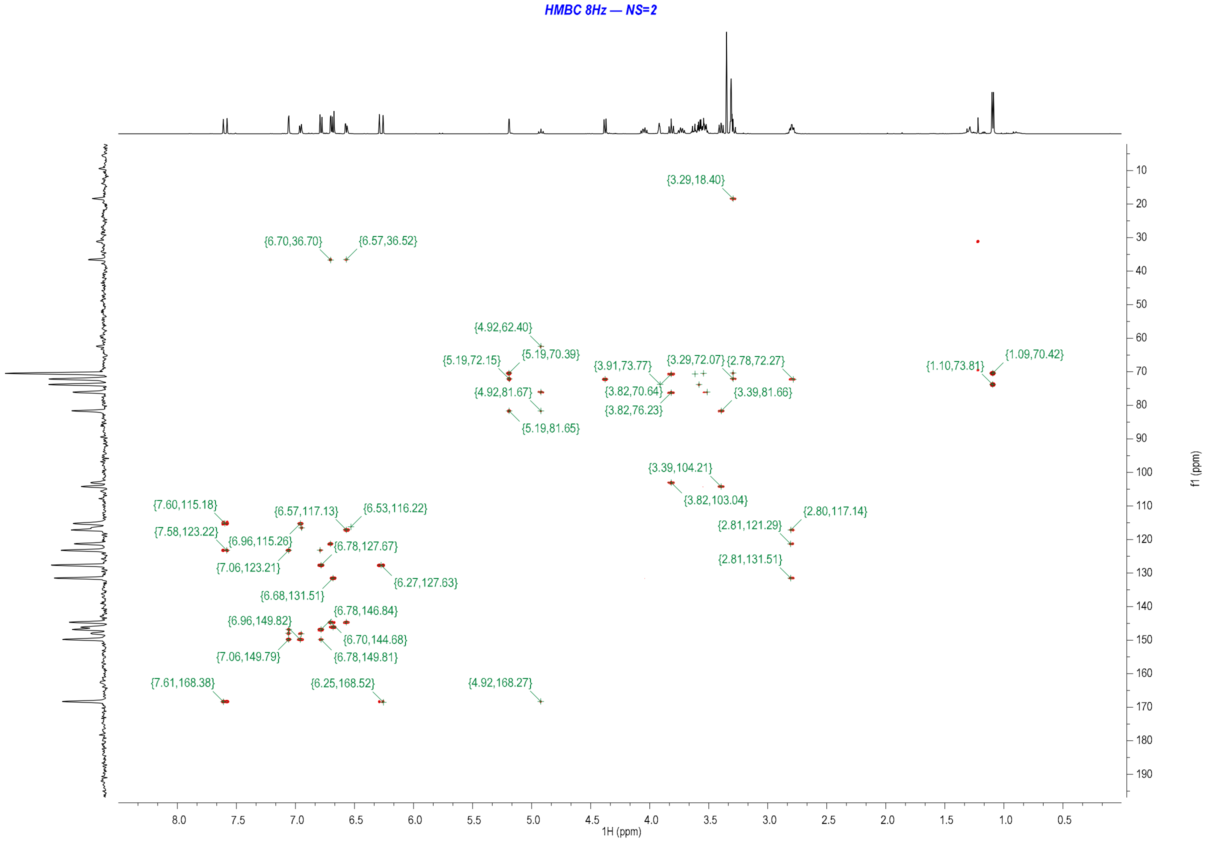


**Figure S4.** $^1$H-$^{13}$C HMBC (8 Hz) NMR spectrum of verbascoside (500/125 MHz, MeOH-$d_4$, 30 °C).

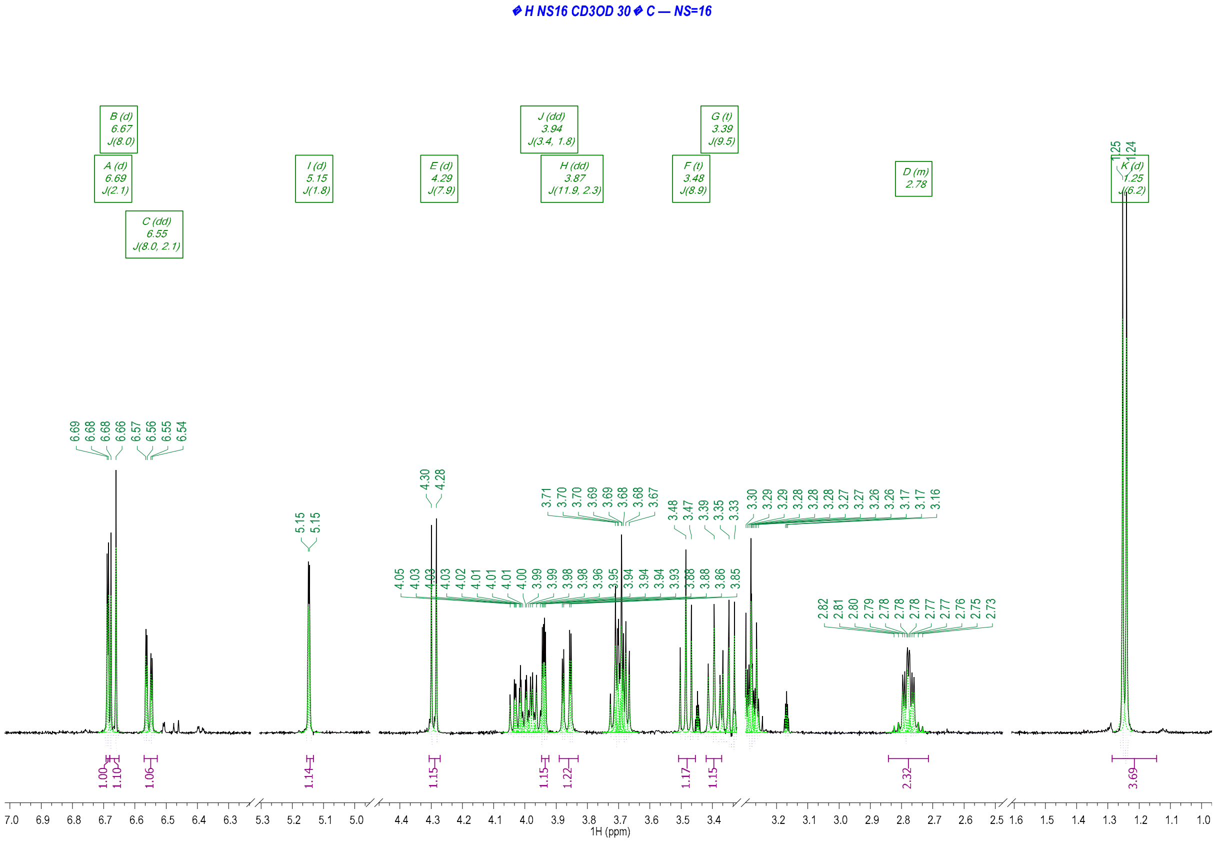


**Figure S5.** $^1$H-NMR spectrum of verbasoside (500 MHz, MeOH-$d_4$, 30 °C).

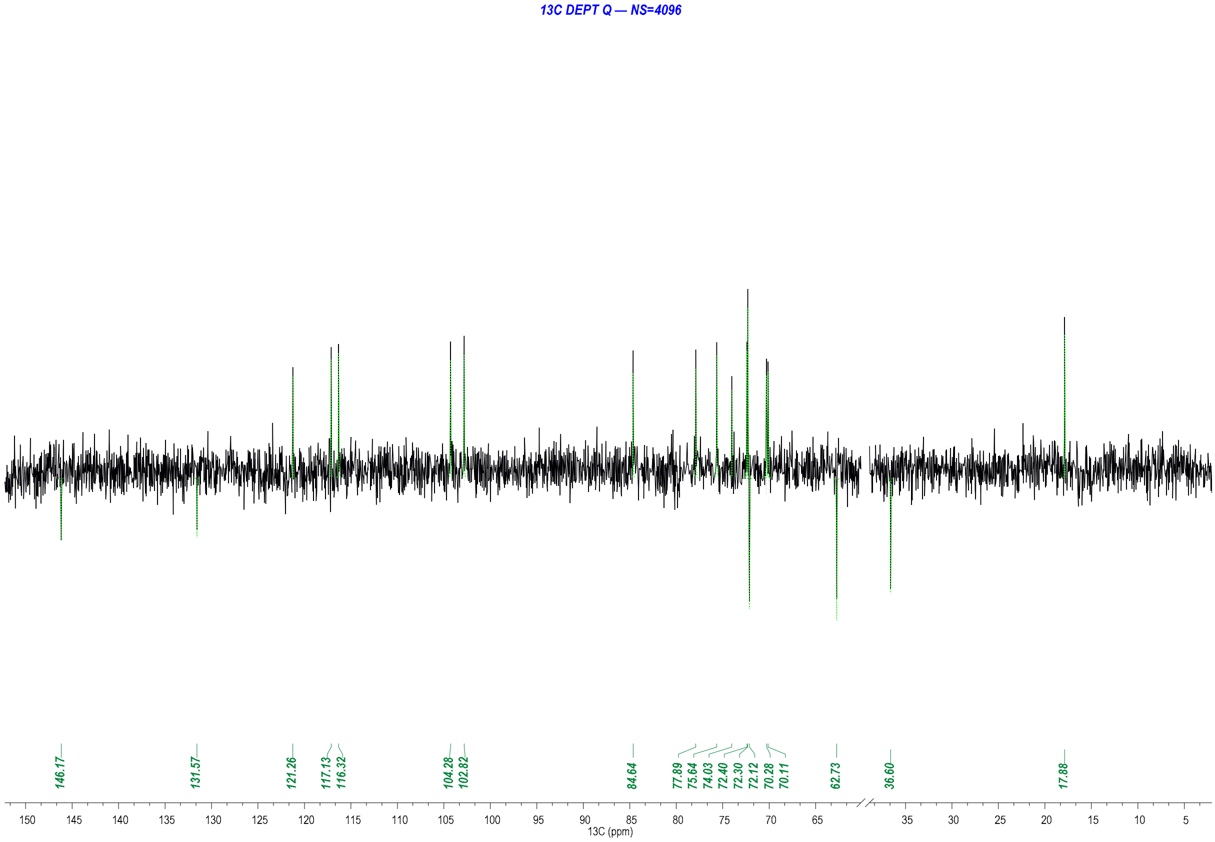


**Figure S6.** $^{13}$C-NMR spectrum of verbasoside (125 MHz, MeOH-$d_4$, 30 °C).

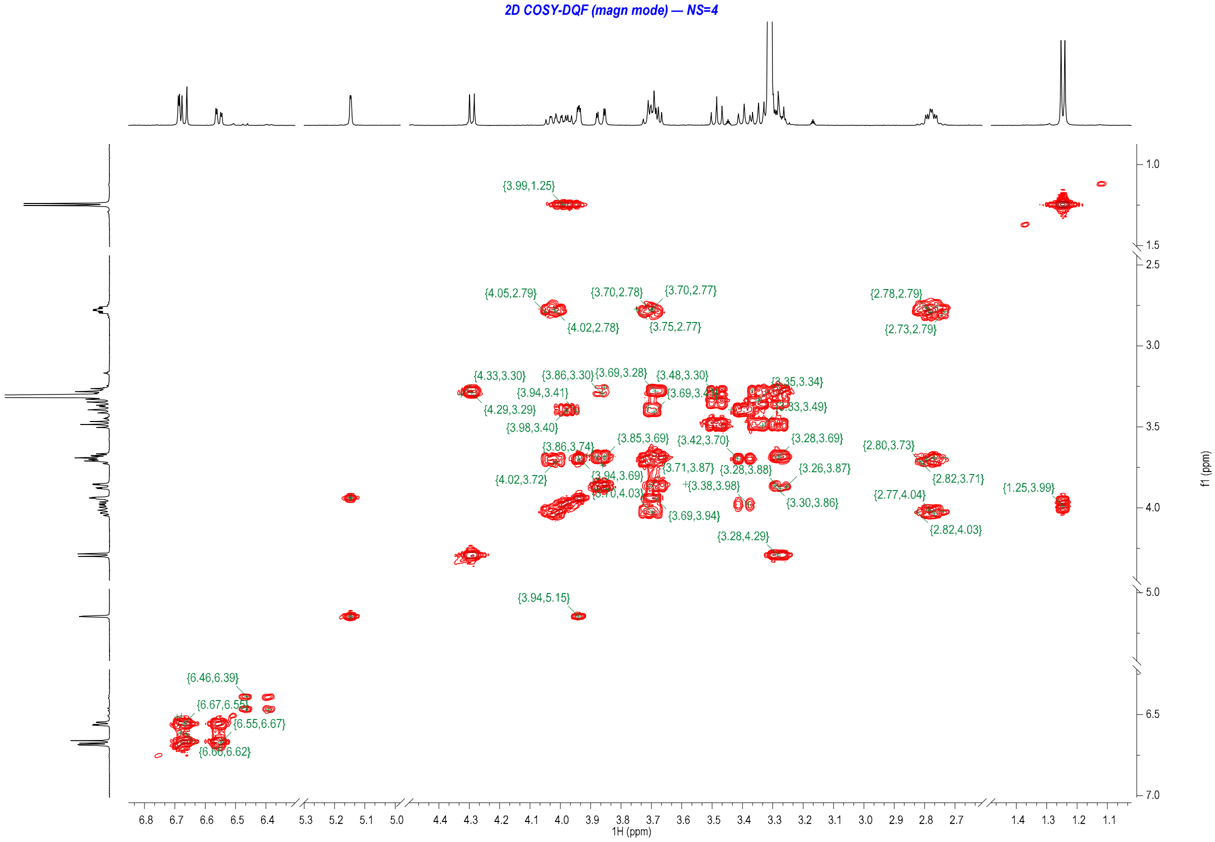


**Figure S7.** $^1$H-$^1$H DQF-COSY NMR spectrum of verbasoside (500 MHz, MeOH-$d_4$, 30 °C).

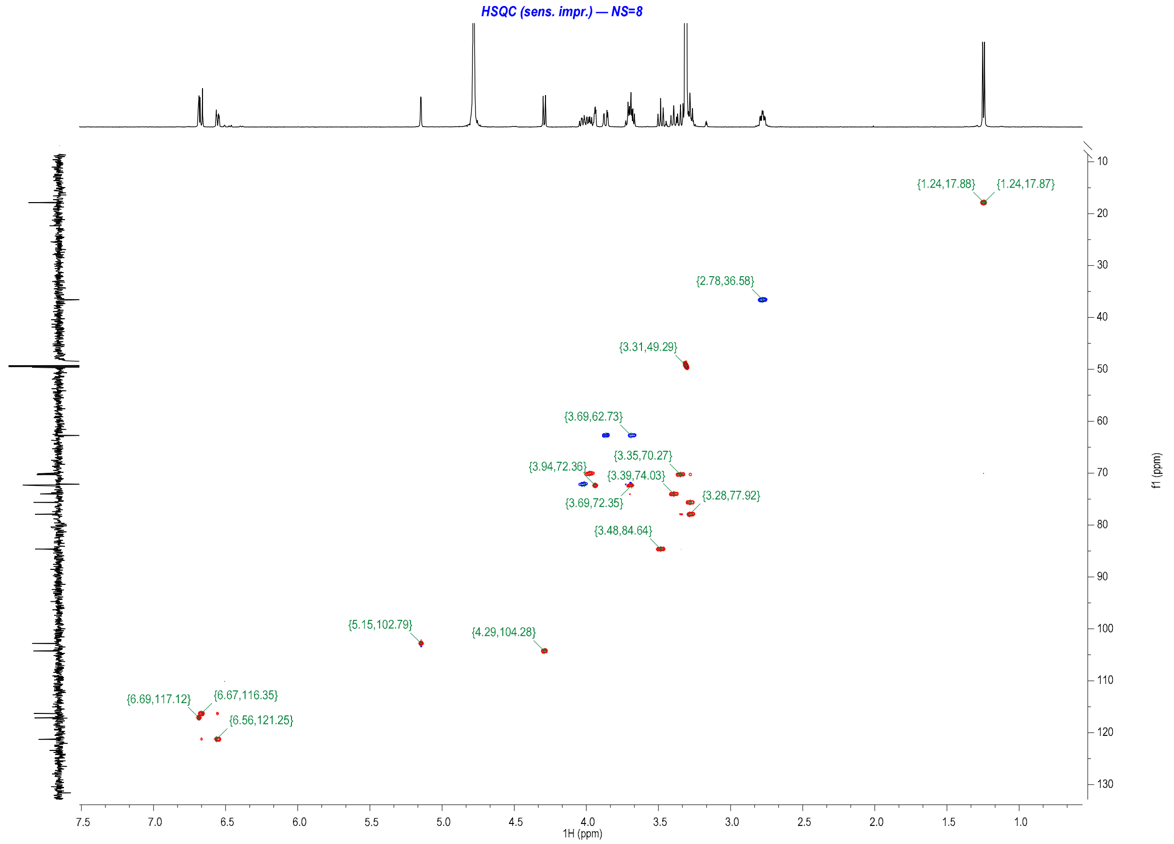


**Figure S8.** $^{1}$H-$^{13}$C HSQC NMR spectrum of verbasoside (500/125 MHz, MeOH-$d_4$, 30 °C).

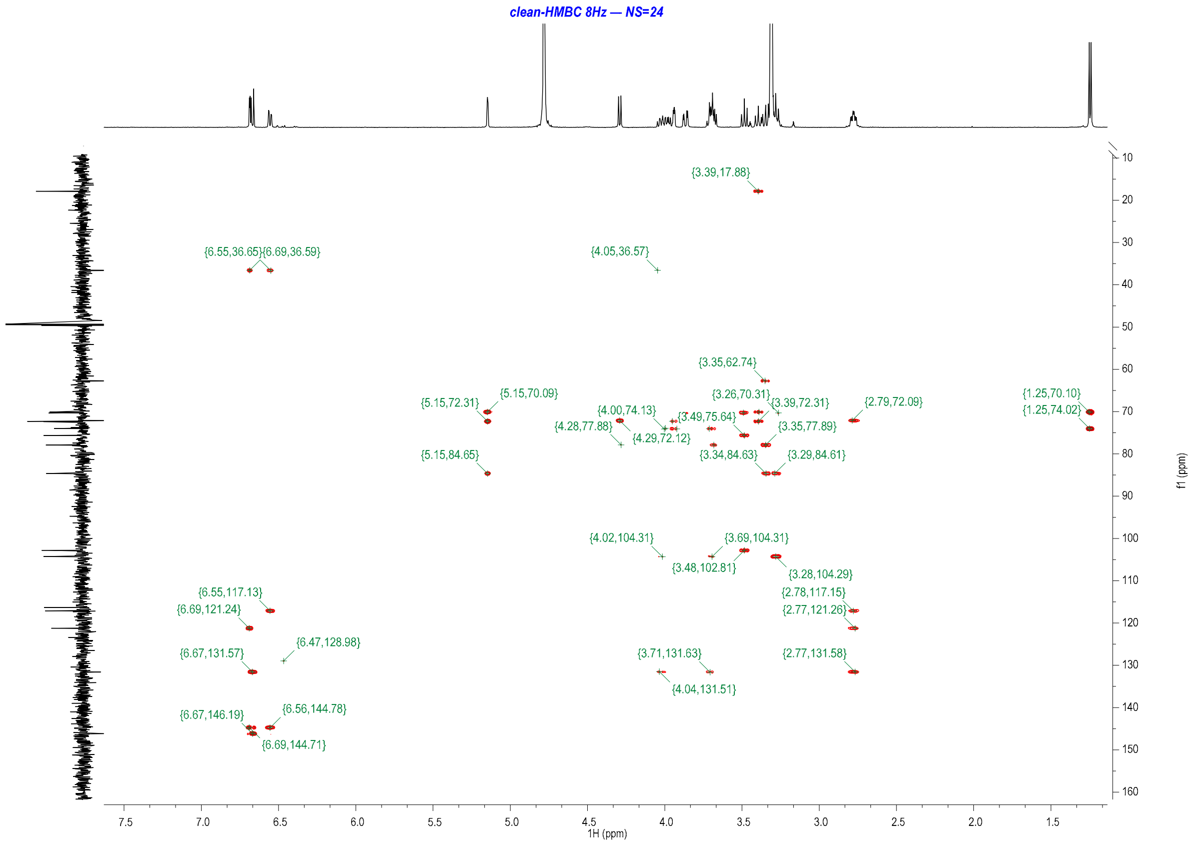


**Figure S9.** $^{1}$H-$^{13}$C HMBC (8 Hz) NMR spectrum of verbasoside (500/125 MHz, MeOH-$d_4$, 30 °C).

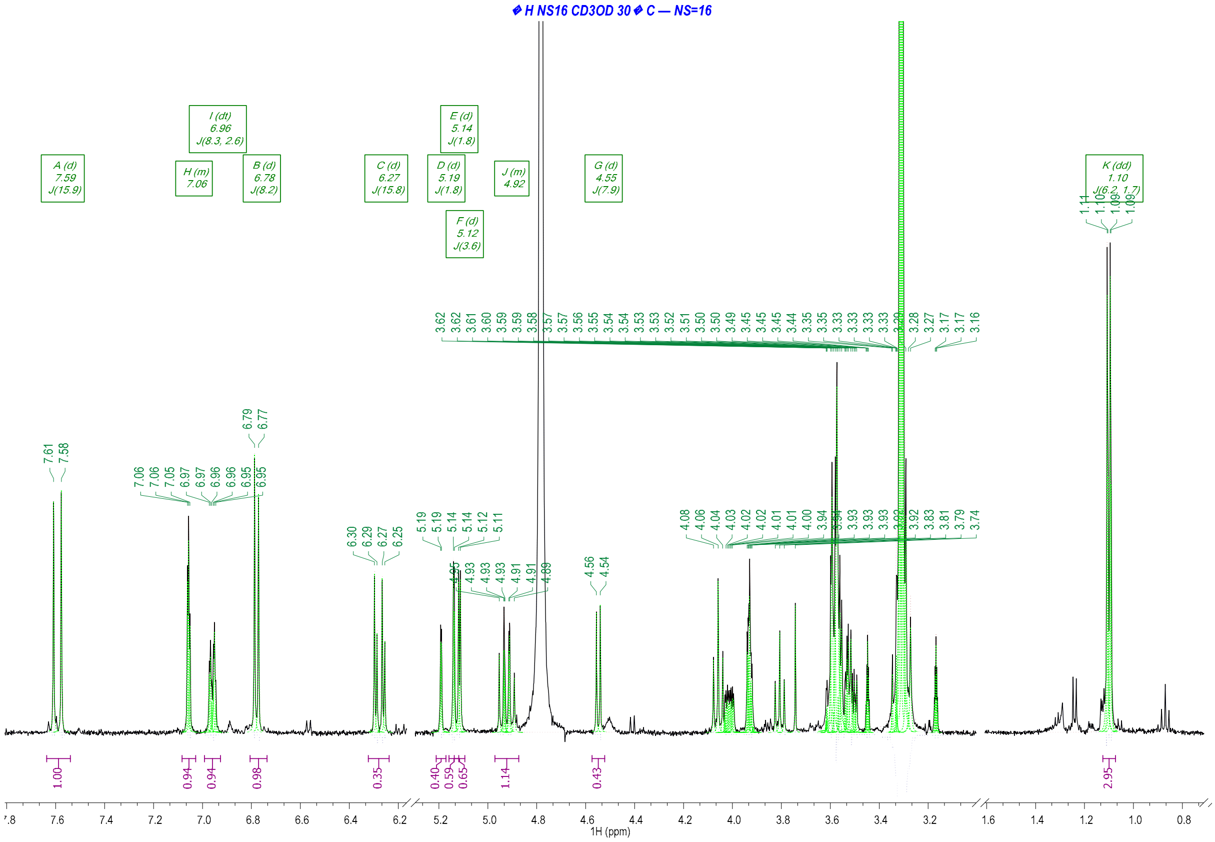

**Figure S10.** $^{1}H$ NMR spectrum of castanoside F (500 MHz, MeOH-$d_4$, 30 °C).

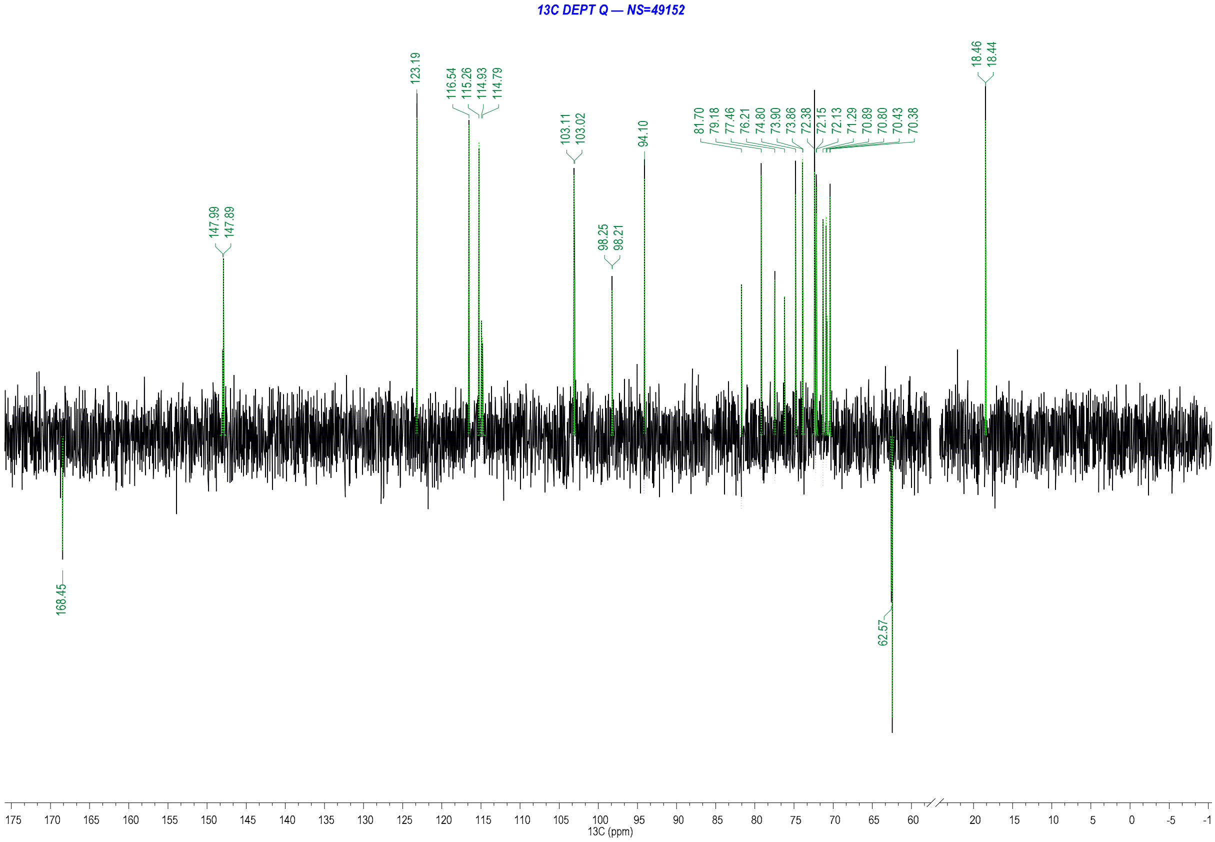

**Figure S11.** $^{13}C$-NMR spectrum of castanoside F (125 MHz, MeOH-$d_4$, 30 °C).

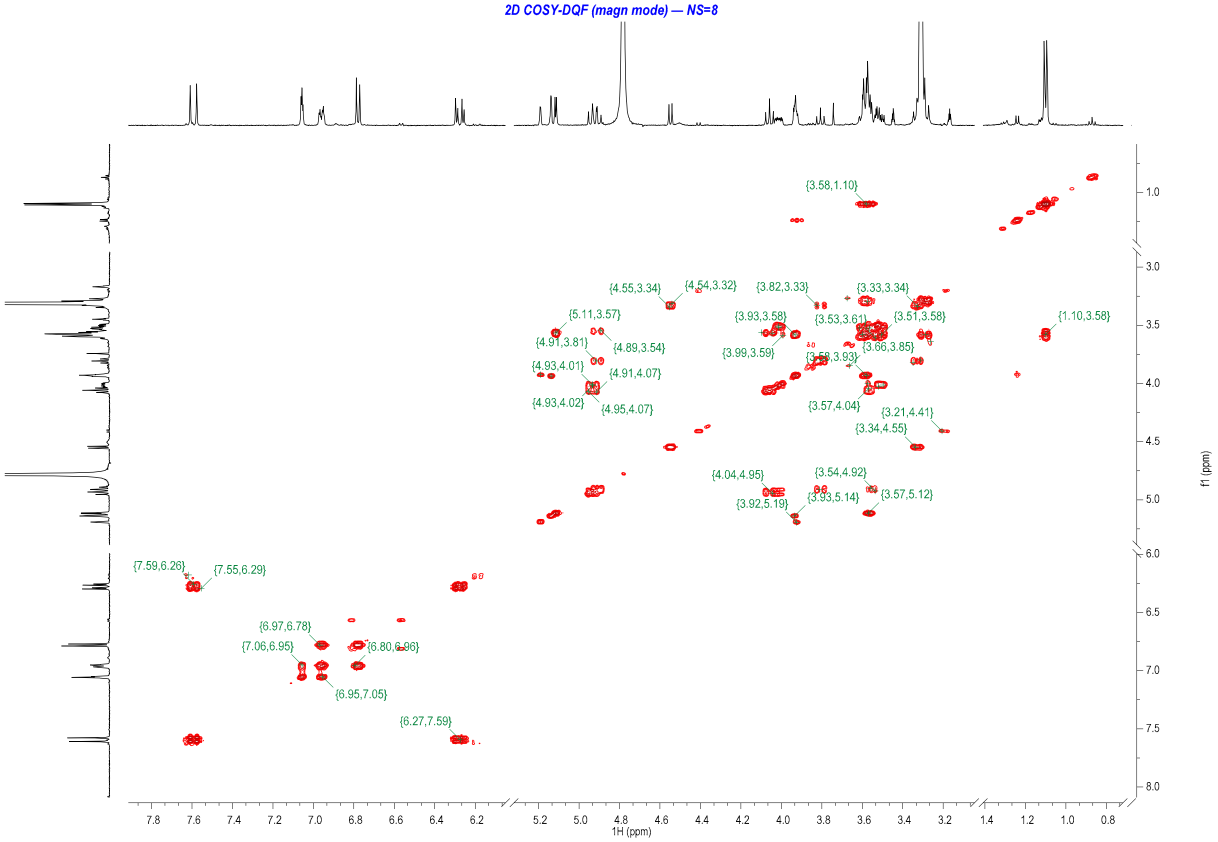


**Figure S12.** $^{1}$H-$^{1}$H DQF-COSY NMR spectrum of castanoside F (500 MHz, MeOH-$d_4$, 30 °C).

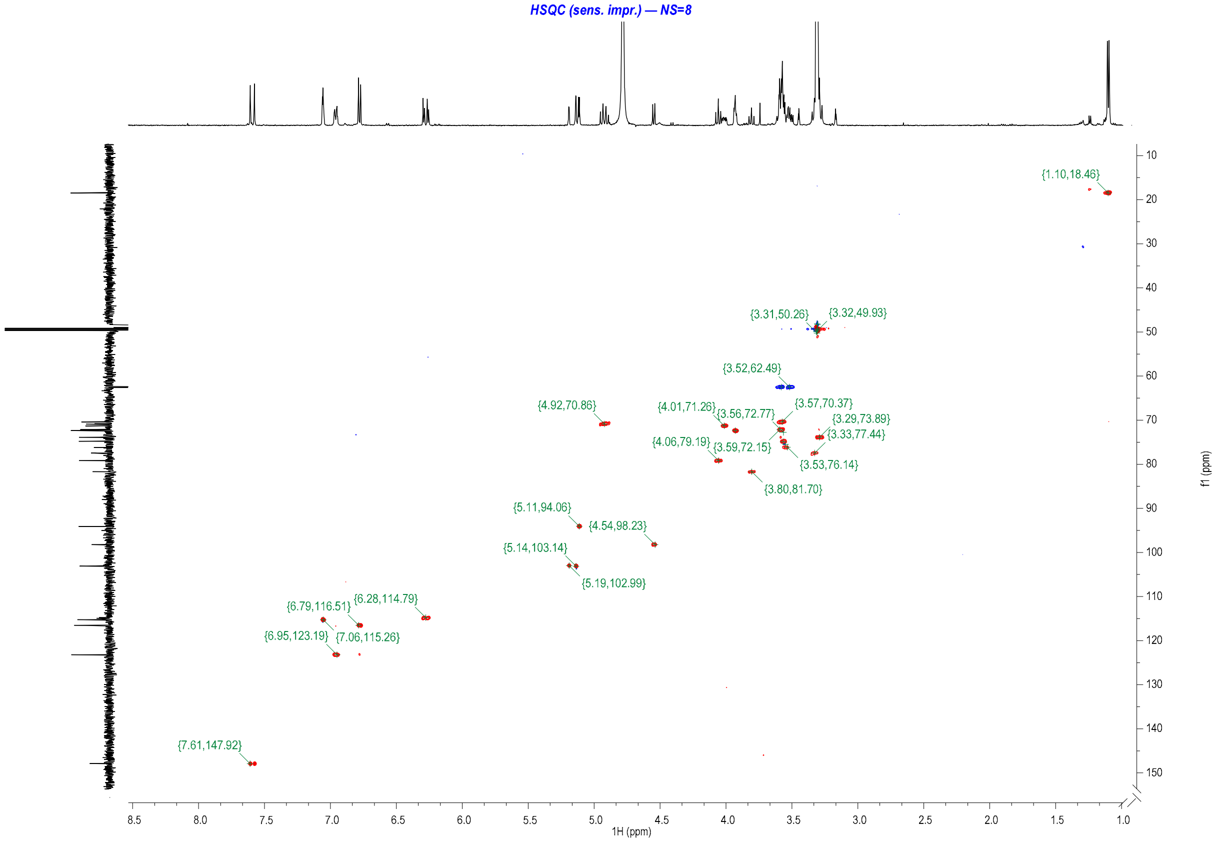


**Figure S13.** $^{1}$H-$^{13}$C HSQC NMR spectrum of castanoside F (500/125 MHz, MeOH-$d_4$, 30 °C).

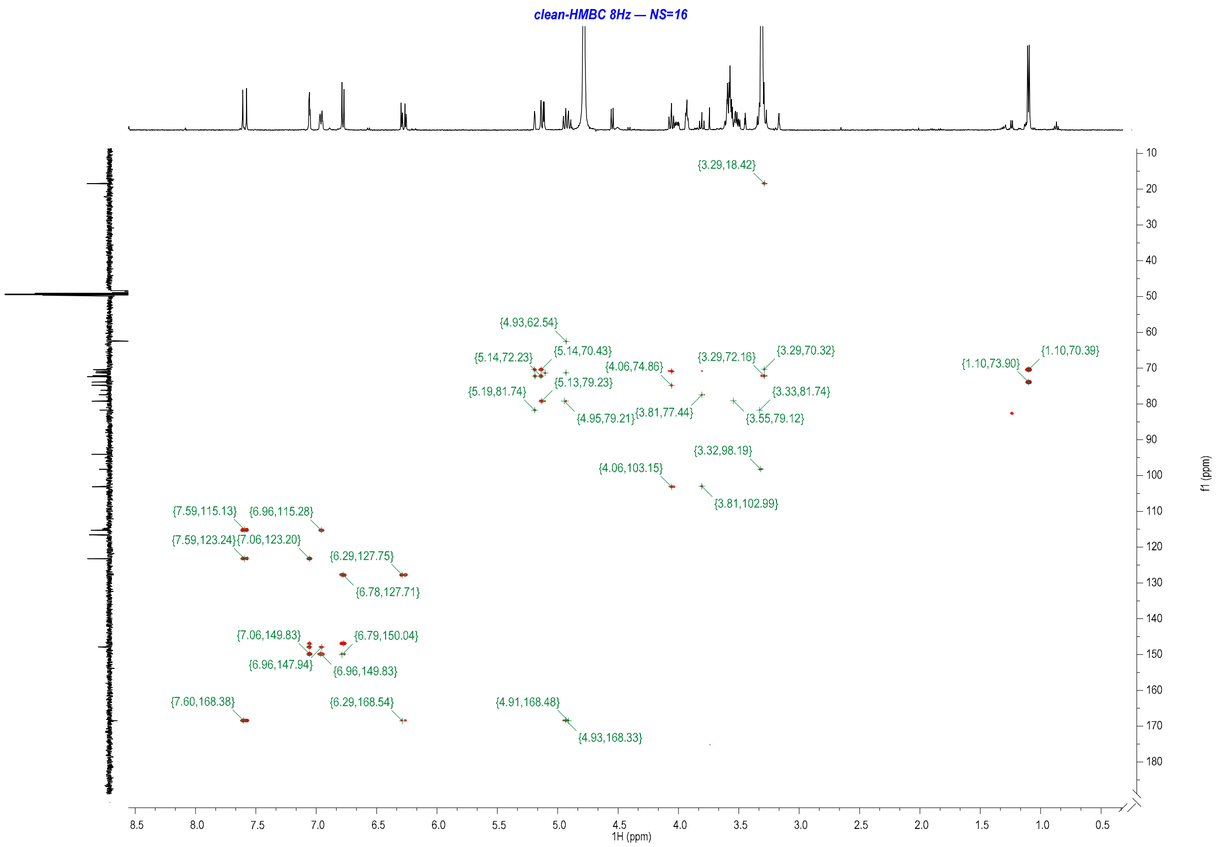


**Figure S14.** $^{1}H$-$^{13}C$ HMBC (8 Hz) NMR spectrum of castanoside F (500/125 MHz, MeOH-$d_4$, 30 °C).

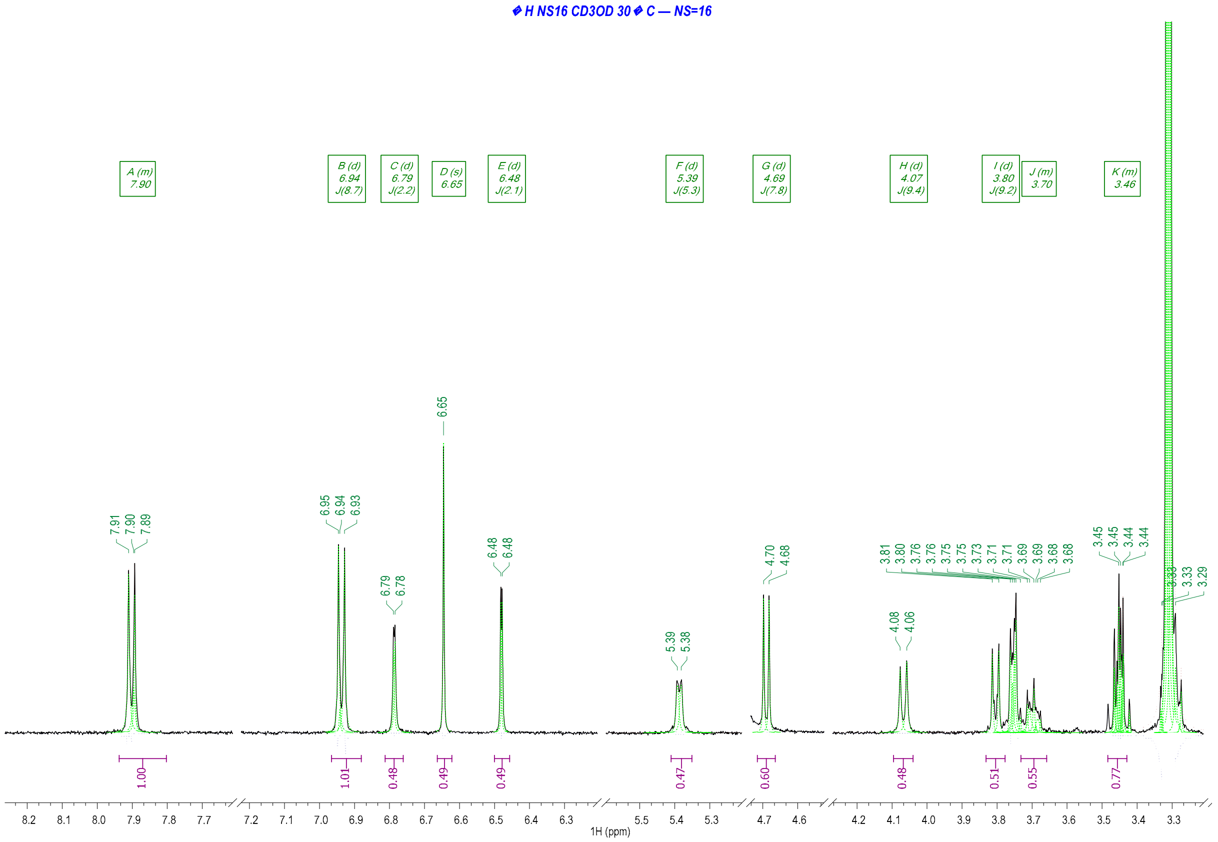


**Figure S15.** $^{1}H$-NMR spectrum of clerodendrin—apigenin 7-*O*-[β-D-glucuronopyranosyl(1→2)-β-D-glucuronopyranoside] (500 MHz, MeOH-$d_4$, 30 °C).

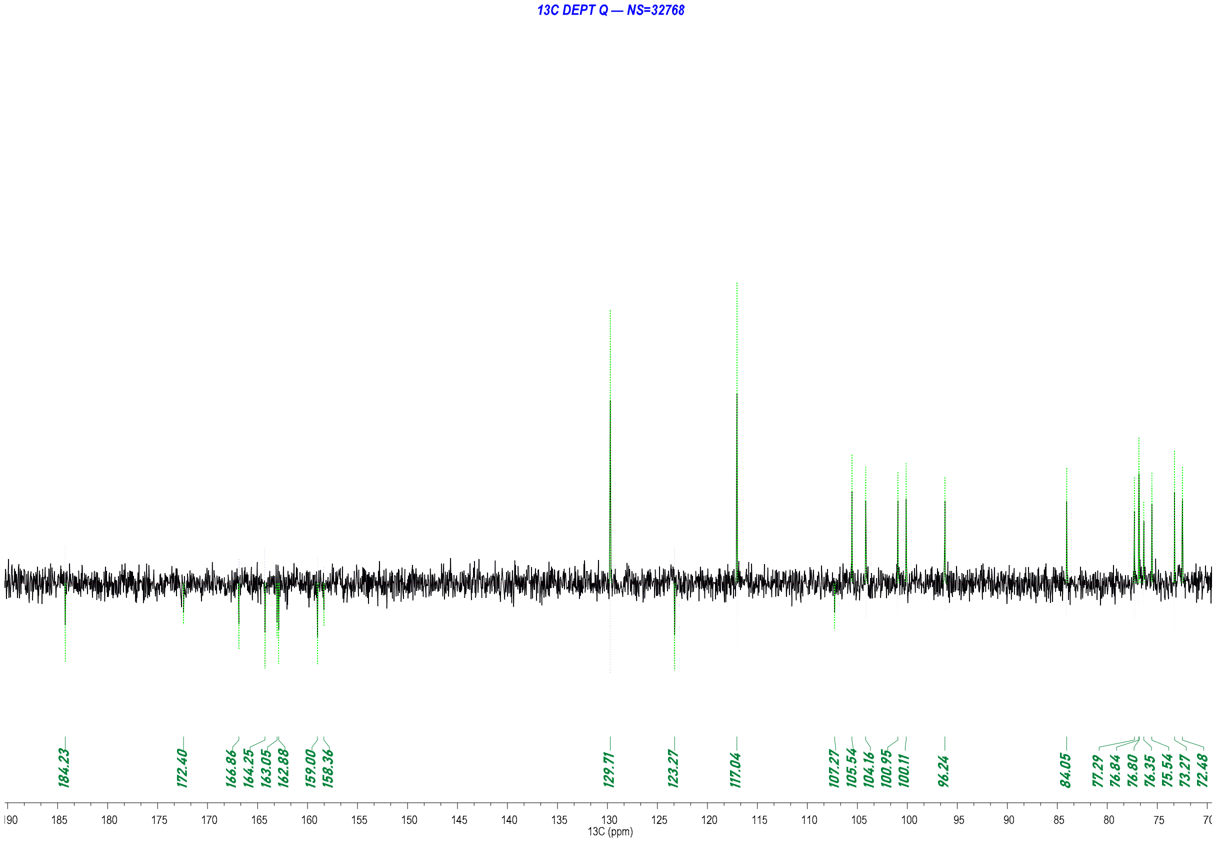


**Figure S16.** $^{13}$C-NMR spectrum of clerodendrin–apigenin 7-*O*-[β-D-glucuronopyranosyl(1→2)-β-D-glucuronopyranoside] (125 MHz, MeOH-$d_4$, 30 °C).

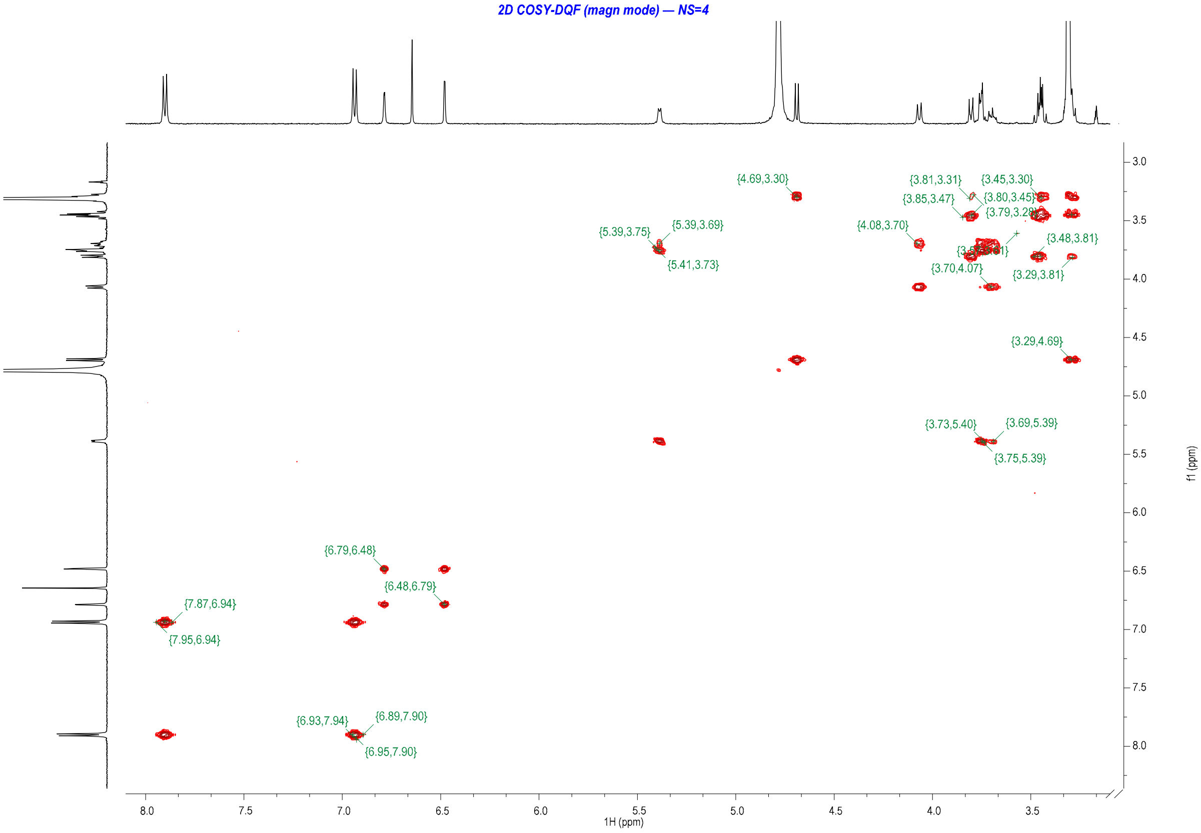


**Figure S17.** $^{1}$H-$^{1}$H DQF-COSY NMR spectrum of clerodendrin – apigenin 7-*O*-[β-D-glucuronopyranosyl(1→2)-β-D-glucuronopyranoside] (500 MHz, MeOH-$d_4$, 30 °C).

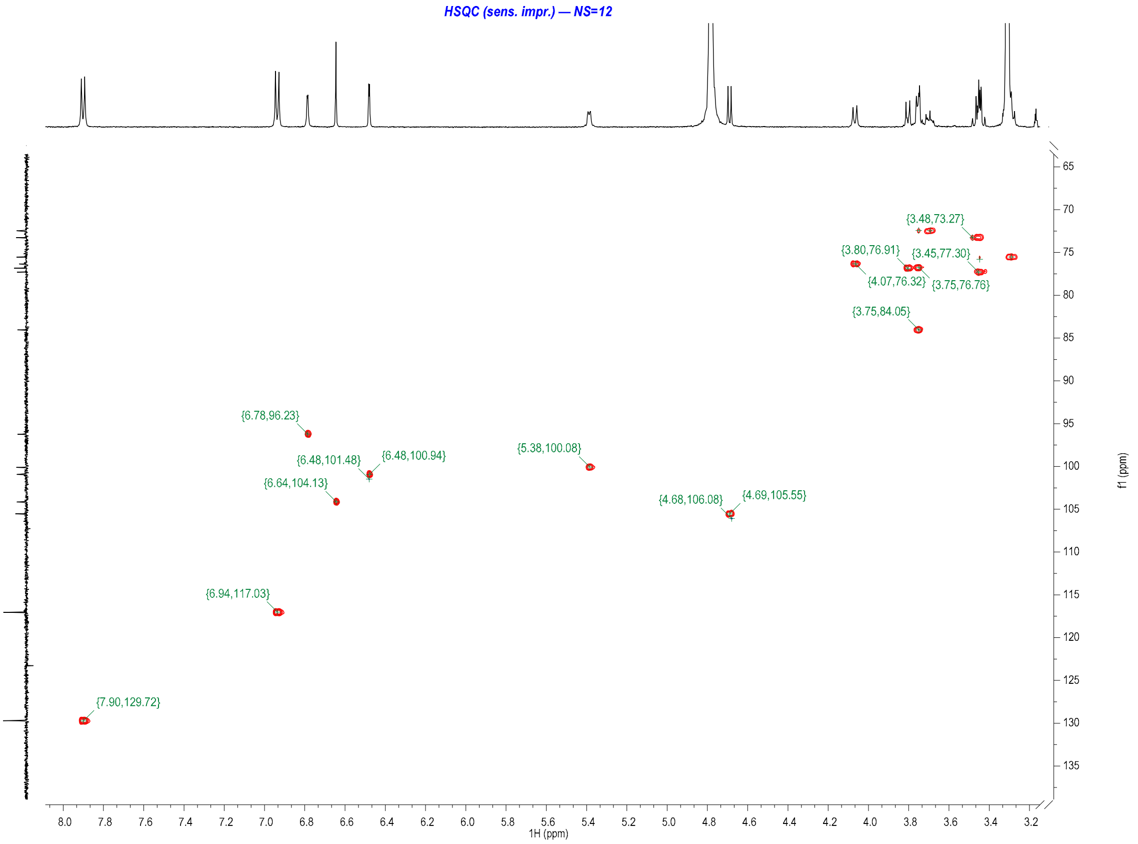


**Figure S18.** $^1$H-$^{13}$C HSQC NMR spectrum of clerodendrin–apigenin 7-*O*-[β-D-glucuronopyranosyl(1→2)-β-D-glucuronopyranoside] (500/125 MHz, MeOH-$d_4$, 30 °C).

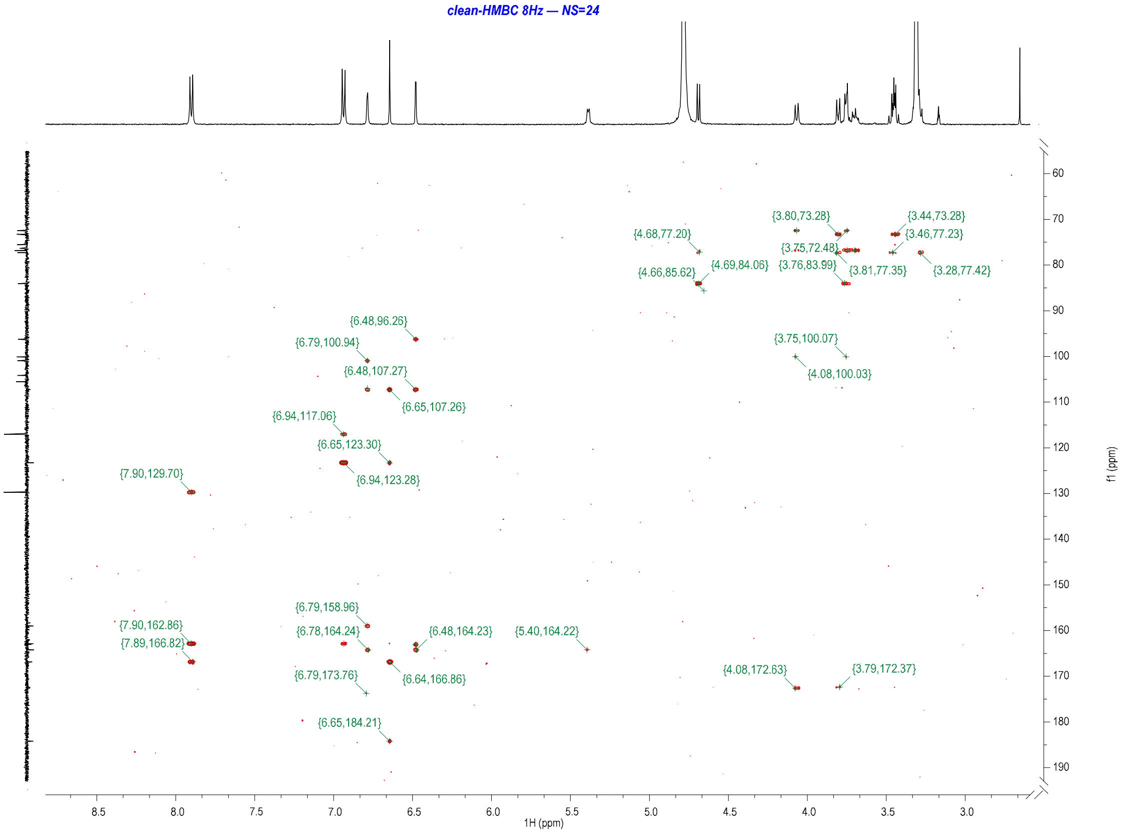


**Figure S19.** $^1$H-$^{13}$C HMBC (8 Hz) NMR spectrum of clerodendrin–apigenin 7-*O*-[β-D-glucuronopyranosyl(1→2)-β-D-glucuronopyranoside] (500/125 MHz, MeOH-$d_4$, 30 °C).

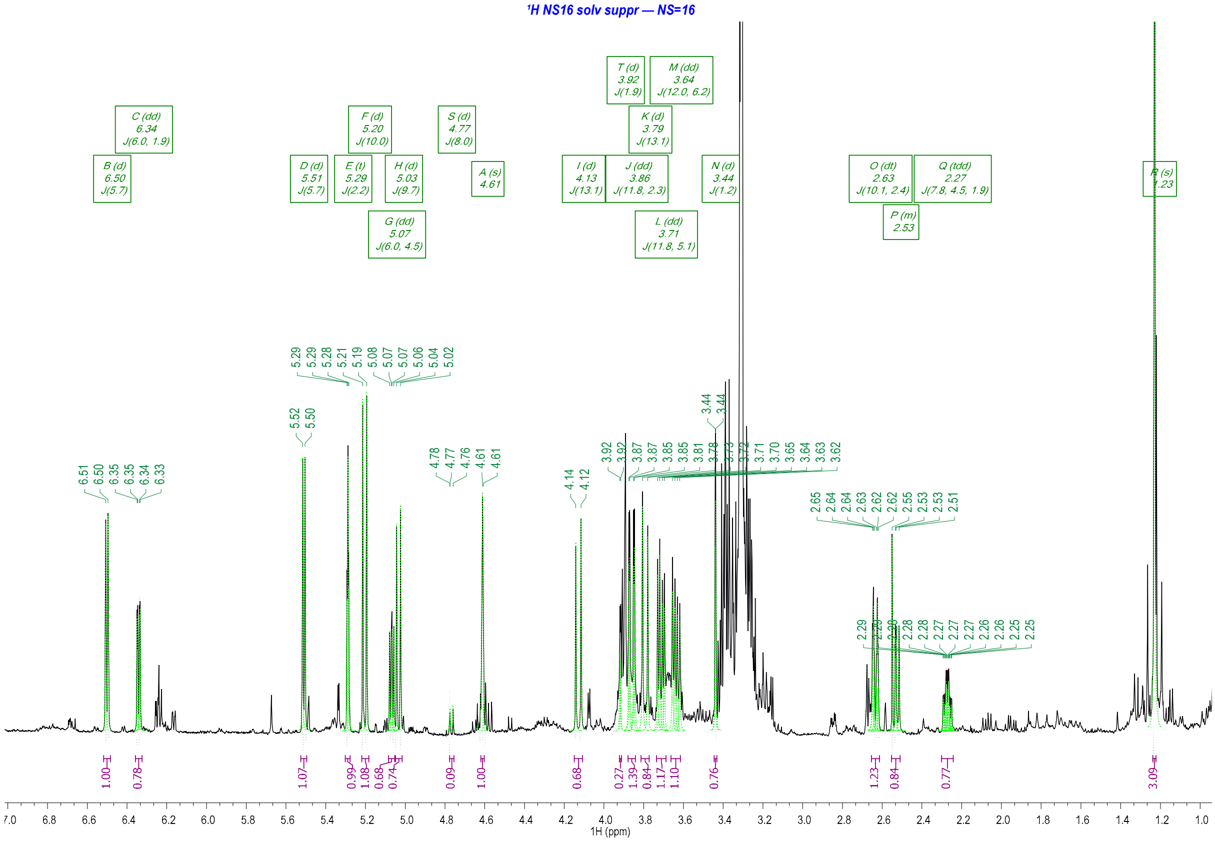


**Figure S20.** $^{1}$H NMR spectrum of catalpol (500 MHz, MeOH-$d_4$, 30 °C).

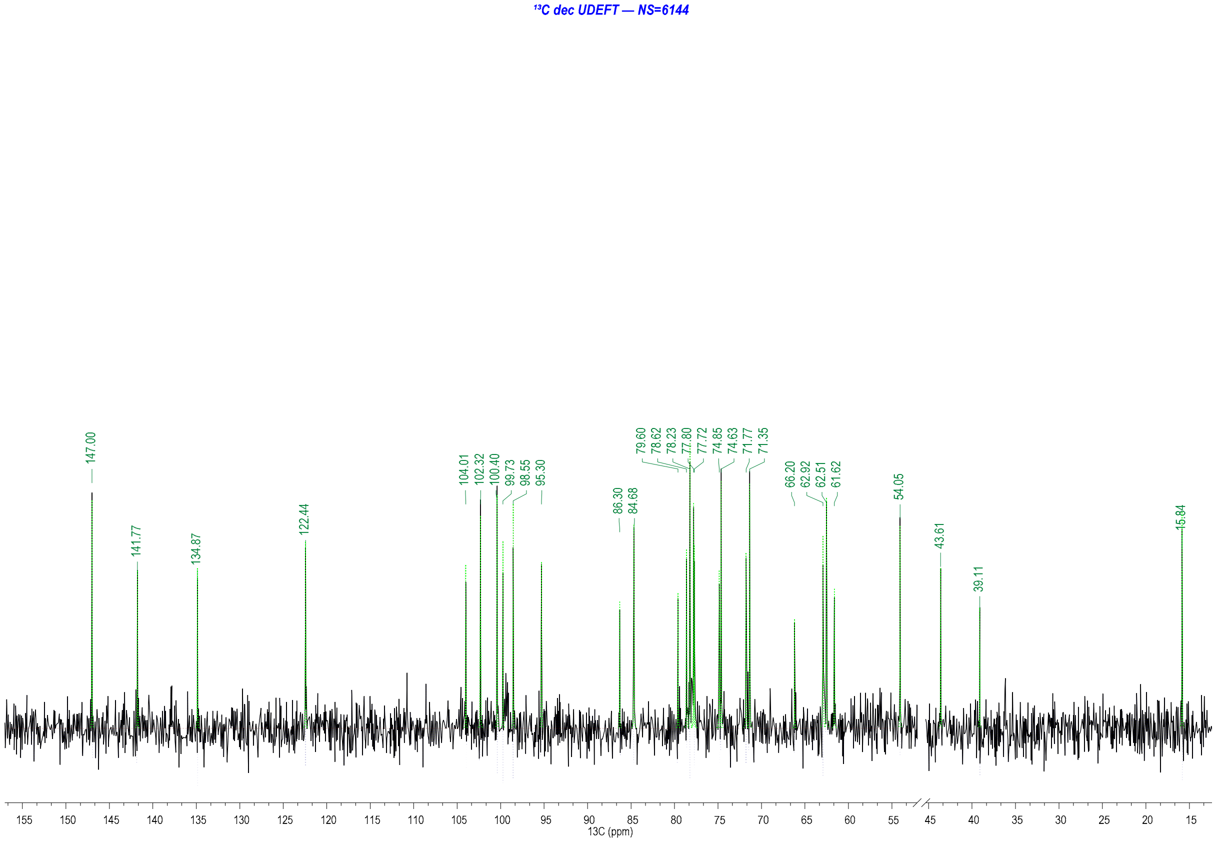


**Figure S21.** $^{13}$C-NMR spectrum of catalpol (125 MHz, MeOH-$d_4$, 30 °C).

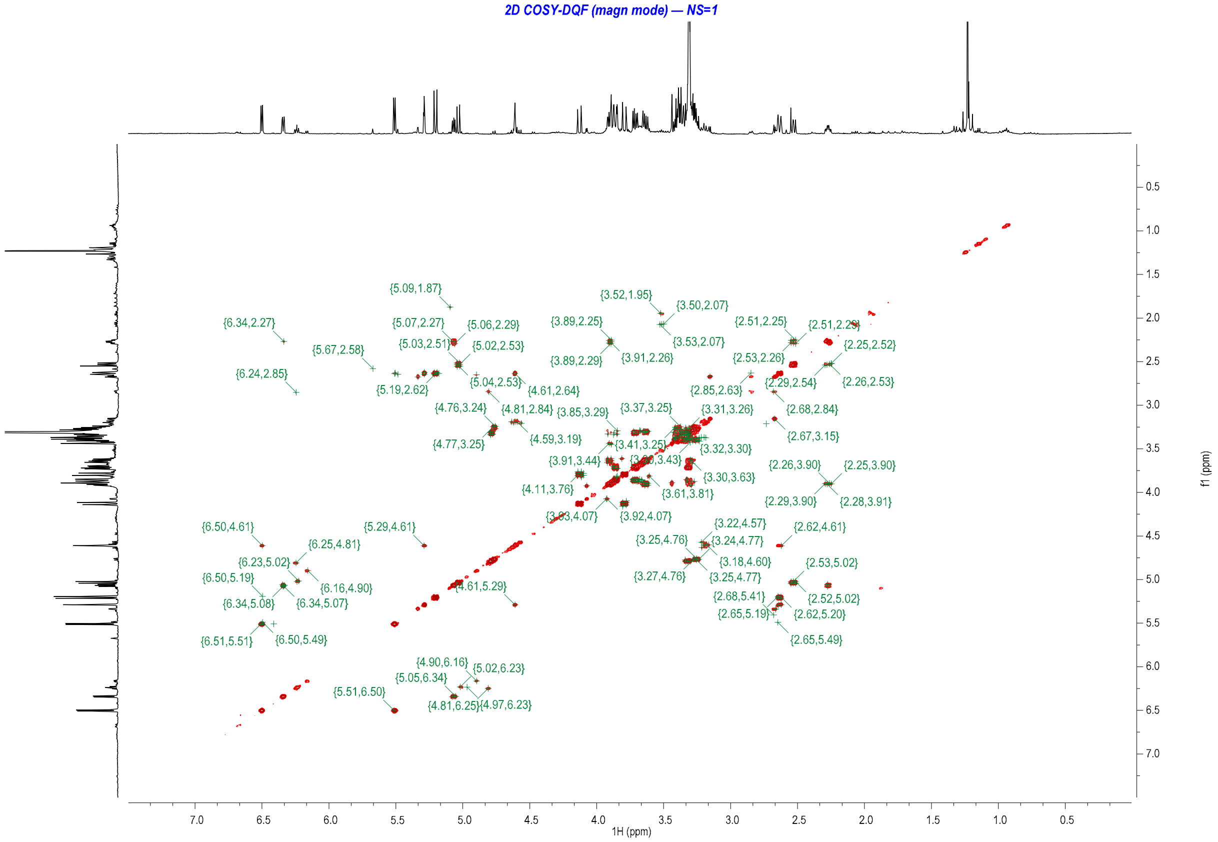

**Figure S22.** $^{1}H$-$^{1}H$ DQF-COSY NMR spectrum of catalpol (500 MHz, MeOH-$d_4$, 30 °C).

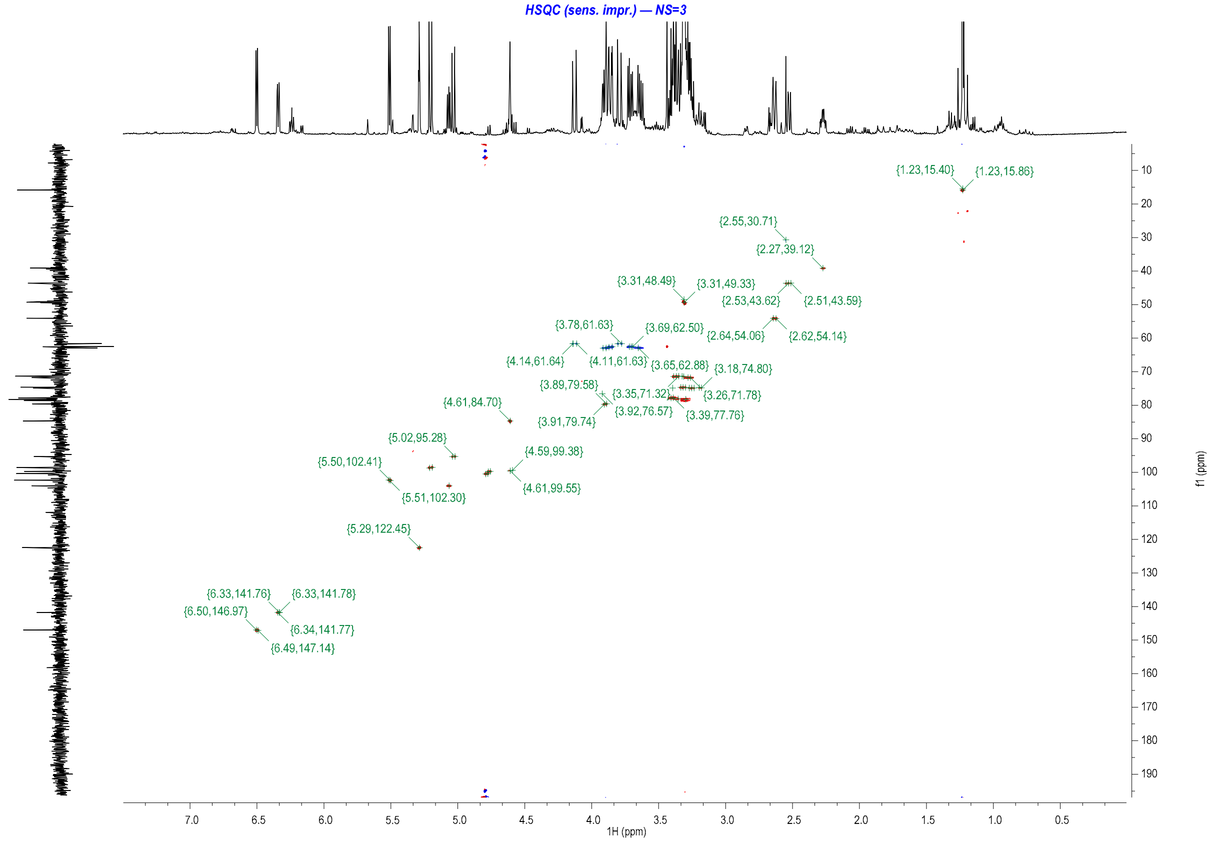

**Figure S23.** $^{1}H$-$^{13}C$ HSQC NMR spectrum of catalpol (500/125 MHz, MeOH-$d_4$, 30 °C).

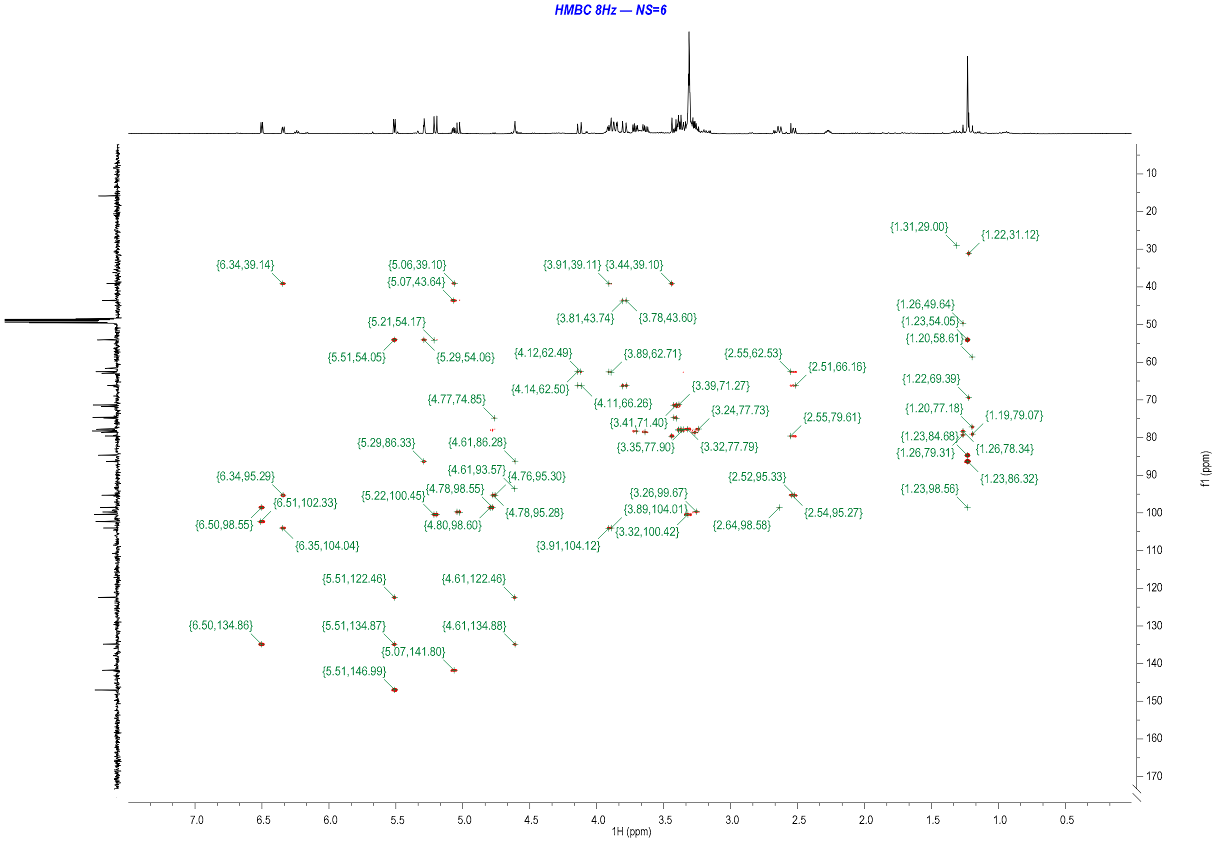


**Figure S24.** $^{1}H$-$^{13}C$ HMBC (8 Hz) NMR spectrum of catalpol (500/125 MHz, MeOH-$d_4$, 30 °C).

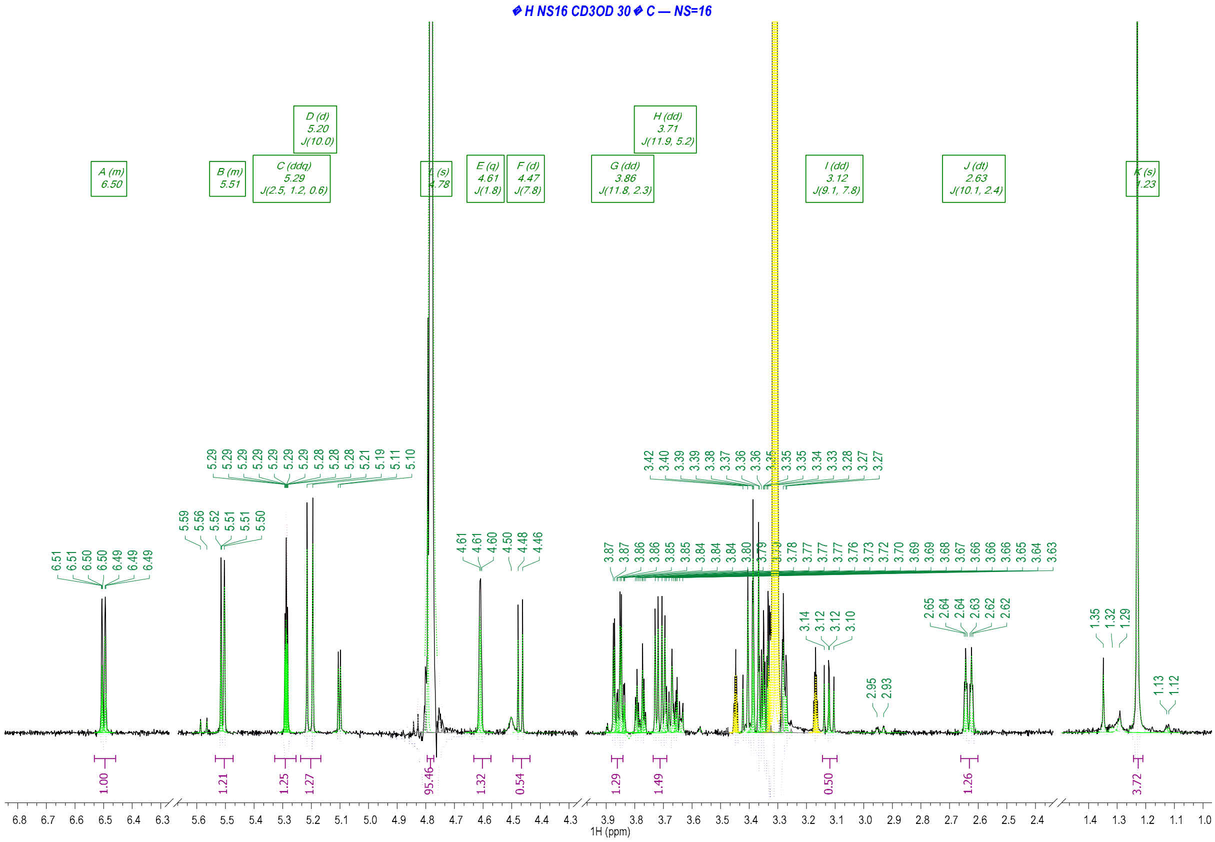


**Figure S25.** $^{1}H$ NMR spectrum of 7-hydroxytomentoside (500 MHz, MeOH-$d_4$, 30 °C).

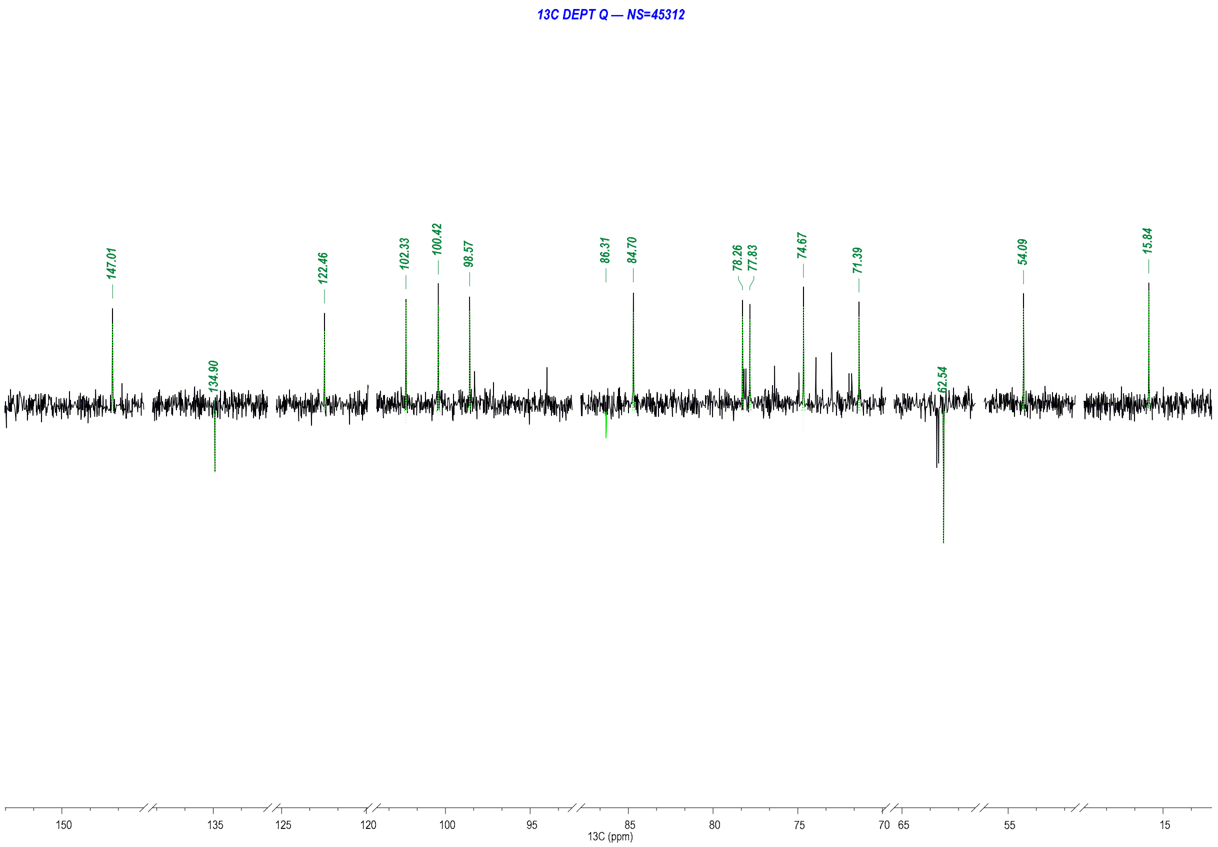


**Figure S26.** $^{13}$C-NMR spectrum of 7-hydroxytomentoside (125 MHz, MeOH-$d_4$, 30 °C).

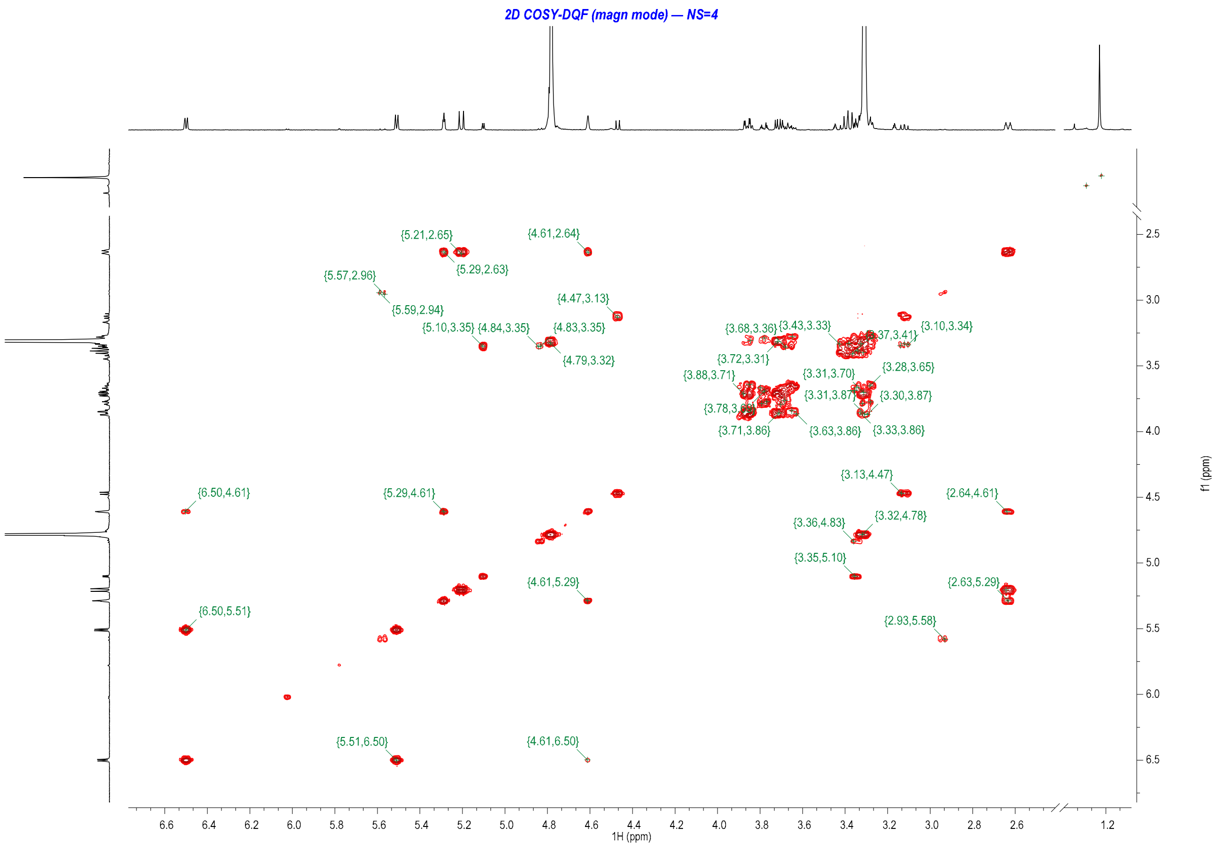


**Figure S27.** $^1$H-$^1$H DQF-COSY NMR spectrum of 7-hydroxytomentoside (500 MHz, MeOH-$d_4$, 30 °C).

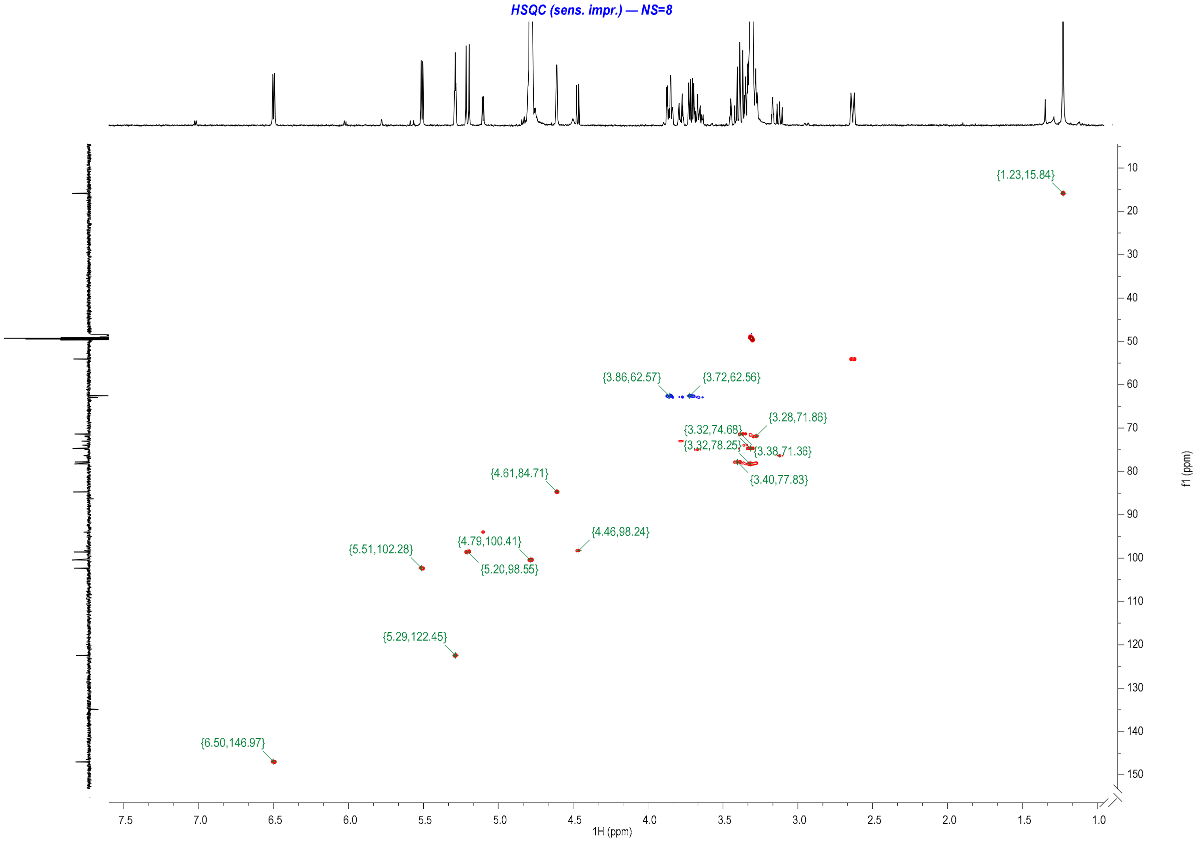


**Figure S28.** $^1$H-$^{13}$C HSQC NMR spectrum of 7-hydroxytomentoside (500/125 MHz, MeOH-*d*$_4$, 30 °C).

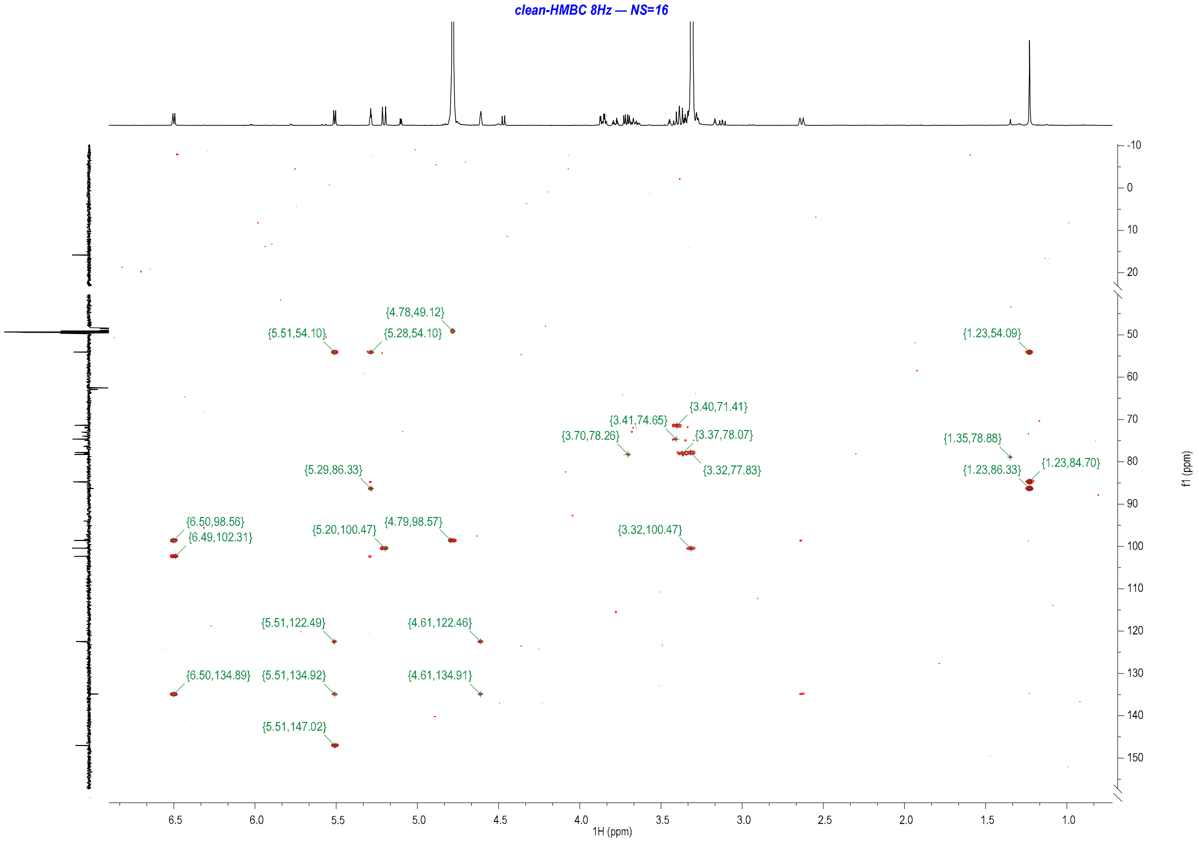


**Figure S29.** $^1$H-$^{13}$C HMBC (8 Hz) NMR spectrum of 7-hydroxytomentoside (500/125 MHz, MeOH-*d*$_4$, 30 °C).
